# An Ex Vivo Evaluation of Air Intrusion Into Pulsed Field Ablation Sheaths During Ablation and Mapping Catheter Insertion

**DOI:** 10.1111/jce.70129

**Published:** 2025-10-02

**Authors:** Chad Gier, Erik Simon, Aamir Ahmed, Graham Peigh, Shivam Patel, Jayson Baman, Aravind Kalluri, Kasen Culler, Kaustubha D. Patil, Anna Pfenniger, Alexandru Chicos, Susan S. Kim, Albert C. Lin, Rod S. Passman, Bradley P. Knight, Nishant Verma

**Affiliations:** ^1^ Division of Cardiology Northwestern University Chicago Illinois USA; ^2^ University of Illinois College of Medicine Chicago Illinois USA; ^3^ Department of Internal Medicine Northwestern University Chicago Illinois USA

**Keywords:** air embolism, air intrusion, atrial fibrillation, pulsed field ablation, sheath management

## Abstract

**Introduction:**

Pulsed field ablation (PFA) is the newest ablation technology, and currently, no data exist on the amount of air intrusion into new, large bore PFA sheaths during ablation or mapping catheter insertion.

**Methods:**

An ex vivo study was performed using various combinations of commercially available PFA ablation catheters and sheaths. Common mapping catheters and a non‐PFA steerable sheath were also evaluated as a reference. The siphon principle was used to create negative pressure to simulate left atrial pressure during spontaneous inspiration. Ablation and mapping catheters were advanced to the end of the sheaths under negative pressure and then removed. Air was withdrawn from the sheaths and was measured in milliliters (mL).

**Results:**

A total of 55 trials were performed. The average volume of air intrusion with all sheath/catheter combinations was 9.6 + 5.2 mL. The 13 Fr (inner diameter) Faradrive sheath (Boston Scientific Inc.) entrained significantly more air (16.5 + 4.1 mL) compared with the 12 Fr FlexCath Contour sheath (Medtronic Inc.) (6.1 + 2.7 mL, *p* < 0.01), 13 Fr Agilis NxT sheath (Abbott Inc.) (8.7 + 1.8 mL, *p* < 0.01), and Vizigo sheath (Johnson & Johnson MedTech Inc.) (5.8 + 2.1 mL, *p* < 0.01), regardless of the catheter used. There was significantly higher volume of air intrusion with the Farawave ablation catheter through the Faradrive sheath (13.6 + 2.0 mL) than through the 13 Fr Agilis (9.4 + 2.1 mL, *p* = 0.03) or the PulseSelect through FlexCath Contour sheath (4.0 + 2.7 mL, *p* < 0.01). Mapping catheters entrained significantly more air than ablation catheters in both the Faradrive (18.0 ± 4.1 mL vs. 13.6 ± 4.1 mL, *p* = 0.04) and the FlexCath Contour (7.1 ± 2.2 mL vs. 4.0 ± 2.7 mL, *p* = 0.03).

**Conclusion:**

Using a model that simulates left atrial PFA in spontaneously breathing patients, a large volume of air intrusion was observed during insertion and removal of ablation and mapping catheters into new, large bore PFA sheaths. New sheath designs are needed to minimize air intrusion during catheter exchanges to avoid air embolism when performing PFA using left atrial delivery sheaths.

## 1 Introduction

Air embolism is a rare but serious complication that can arise during left sided cardiac procedures requiring transseptal catheterization, including atrial fibrillation (AF) ablation. Air embolism may result in devastating neurologic events, systemic embolization, right coronary artery occlusion, or death [1, 2, 3]. Guidelines quote incidence of less than 1% but studies have reported rates of air embolism as high as 2.6% of AF ablation cases [4, 5]. Many procedural aspects may contribute to the formation of air emboli, including multiple catheter and sheath exchanges, improper infusion management, and negative left atrial (LA) pressure during inspiratory phases of the respiratory cycle [6, 7, 8].

There is heightened awareness of air intrusion into sheaths, as some pulsed field ablation (PFA) sheaths are now designed with transparent shafts, allowing for direct visualization of large air bubbles during catheter insertion or exchange [9].

Previous studies have evaluated air intrusion during catheter insertion into older generations of ablation sheaths; however, the larger size of PFA ablation catheters has necessitated the development of new, larger bore sheaths [10, 11]. To date, there is no data on the amount of air intrusion into these newly developed sheaths. Therefore, we performed an ex vivo study to evaluate the amount of air intrusion into PFA sheaths during ablation or mapping catheter insertion of commercially available PFA sheaths.

## 2 Methods

An investigator‐initiated ex vivo study was performed using simulated negative pressure to assess air intrusion into PFA steerable sheaths during catheter insertion and removal. This study did not involve human subjects and was therefore exempt from Institutional Review Board (IRB) review.

### 2.1 Negative Pressure

**Figure 1:**
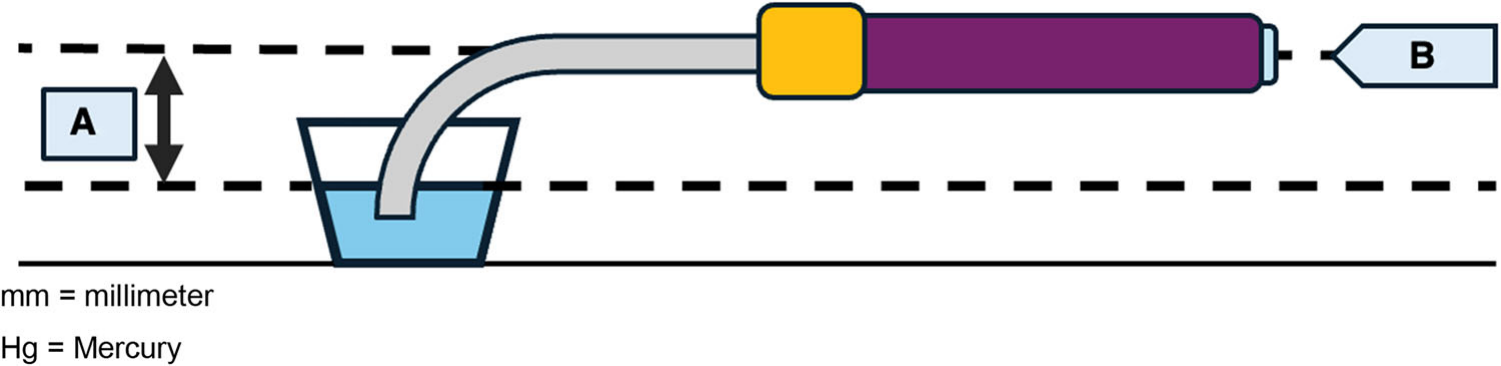
Illustration of experimental design. (A) The height from the water surface to the bottom of the steerable sheath, which corresponded to 272 mm to create a negative pressure of −20 mmHg. (B) A mapping or ablation catheter was inserted into the sheath, advanced to the end, and removed. Air was then withdrawn from the sidearm of the sheath. The type of catheter was changed, and the process was repeated. mm, millimeter; Hg, mercury.

Although LA pressure is usually positive, studies have shown that LA pressure is commonly negative during inspiration when patients are under moderate sedation and can be greater than −13 mmHg in a large proportion of patients [12]. To improve sensitivity and simulate maximal‐risk conditions, a negative pressure of −20 mmHg was applied to the sheath using the siphon principle. Based on previously published methods, negative pressure was created by filling the inside of a reservoir and sheath with water and raising the end of the sheath to 272 mm (1 mmHg corresponds to 13.6 mm at the height of water) [10] (Figure 1).

### 2.2 Sheath and Catheter Preparation, Insertion, and Removal

All sheaths and catheters were prepared in accordance with the manufacturers' specifications, including flushing, sheath deflection, dilator insertion, and removal. A total of four sheaths and four catheters were evaluated. At the time of the study, three commercially available PFA sheaths—including the 13 Fr (inner diameter) Faradrive™ (Boston Scientific), 12 Fr FlexCath Contour™ (Medtronic), and 13 Fr Agilis™ NxT (Abbott)—were available in the United States. The non‐PFA specific 8.5 Fr Vizigo (Johnson & Johnson MedTech) sheath was also evaluated. Four catheters were evaluated, including two PFA catheters (Farawave™ [Boston Scientific] and PulseSelect™ [Medtronic]) and two mapping catheters (Advisor™ HD Grid [Abbott] and Octaray™ [Johnson & Johnson MedTech]). Each ablation catheter was tested with its manufacturer‐specific sheath, with the Farawave additionally trialed in the 13 Fr Agilis NxT. Both mapping catheters were tested in all sheaths. Specifications for each sheath and catheter can be found in Tables 1A and 1B.

**Table 1A:** Comparison of sheath specifications.

	Faradrive [16]	FlexCath Contour [17]	13 Fr Agilis NxT [18]	Vizigo [19]
Inner diameter (Fr)	13	12	13	8.5
Outer diameter (Fr)	16.8	16	17.2	11.5
Usable length (cm)	74	65.5	71	71
Transparent shaft?	Yes	No	No	No
Bidirectional?	No	Yes	Yes	Yes
Hemostatic valve opening shape	Cat‐eye	Asterisk	Round, recessed	Asterisk

*Note:* This table provides an overview of the key design characteristics of the ablation sheaths evaluated in this study. It highlights differences in diameter, valve design, and structural features.

**Table 1B:** Comparison of catheter specifications.

	Farawave [16]	PulseSelect [20]	HD Grid [21]	Octaray [22]
Type	Ablation	Ablation	Mapping	Mapping
Shaft diameter (Fr)	12	9	8	8
Array diameter (cm)	3.1/3.5	25	1.3	3.0/4.0
Usable length (cm)	115	99	105	113
Electrodes	20	9	16	48
Introducer?	No	Yes	Yes	Yes
Catheter tip shape	Pentaspline	Lasso	Grid	Octapoles

*Note:* This table provides an overview of the key design characteristics of the ablation and mapping catheters evaluated in this study. It highlights differences in size, presence of introducer tool, and catheter tip shape.

While maintaining the sheath at the prespecified height, catheters were inserted and advanced until the tip of the catheter was visible at the end of the sheath. To mimic standard clinical practice, if a catheter had an introducer tool, this column was filled with water before catheter insertion. Air was withdrawn from the side port after catheter insertion and measured to the nearest milliliter (mL). The catheters were then removed. Air was, again, withdrawn from the side port and measured to the nearest mL. These two values were added together and became the total air intrusion measurement for each trial. Catheters were inserted and removed at a consistent pace, each taking approximately 10 s. Five trials were performed for each combination of catheter and sheath. The catheters and sheaths were not connected to continuous infusions to avoid perturbation of the negative pressure created in the model.

## 3 Statistical Analysis

Statistical analyses were performed using SPSS version 30 (IBM Corp.). Continuous variables were examined using the Shapiro–Wilk test for normality. Most continuous variables had normal distribution; therefore, data were presented as means. Comparisons between groups were performed using one‐way analysis of variance (ANOVA). Bonferroni correction for post hoc pairwise comparisons was used when comparing more than two groups. Figures were created with Prism 10 (GraphPad Software). Data are presented as mean + standard deviation. The *p* values were two‐sided with a value less than 0.05 considered statistically significant.

## 4 Results

A total of 55 trials were performed (11 combinations, 5 trials each). The mean volume of air intrusion with all sheath/catheter combinations was 9.6 + 5.2 mL.

### 4.1 Comparing Sheaths

**Figure 2:**
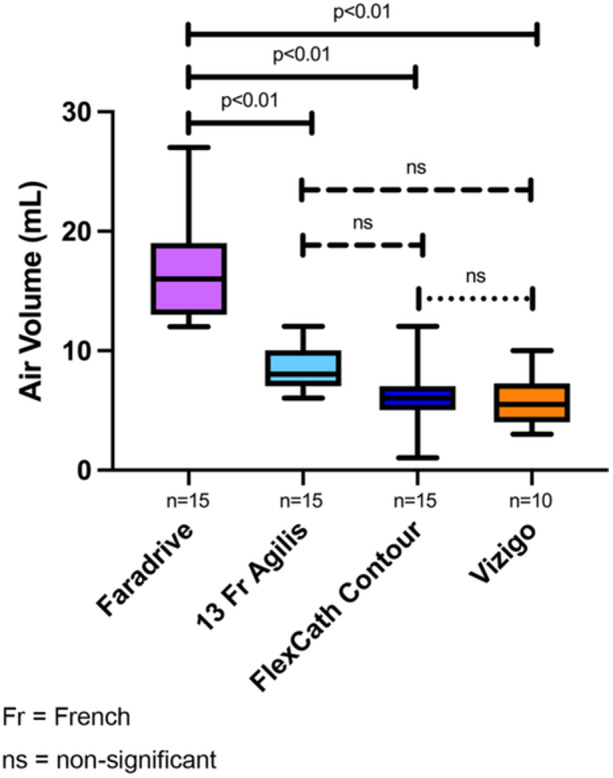
Box plots comparing average air intrusion volumes by sheath, including all catheters tested. Each box plot represents the distribution of air intrusion volumes associated with a specific sheath, incorporating data from both mapping and ablation catheters. The highest average volume of air intrusion was seen with the Faradrive and was significantly higher than the 13 Fr Agilis NxT, FlexCath Contour, and Vizigo. Fr, French; ns, nonsignificant.

Overall, when combining all trials, the highest volume of air intrusion was seen in the Faradrive sheath (16.5 + 4.1 mL) and was significantly more than the FlexCath Contour (6.1 + 2.7 mL, *p* < 0.01), 13 Fr Agilis (8.7 + 1.8 mL, *p* < 0.01), and Vizigo (5.8 + 2.1 mL, *p* < 0.01) as shown in Figure 2.

### 4.2 Comparing PFA “Systems”

**Figure 3:**
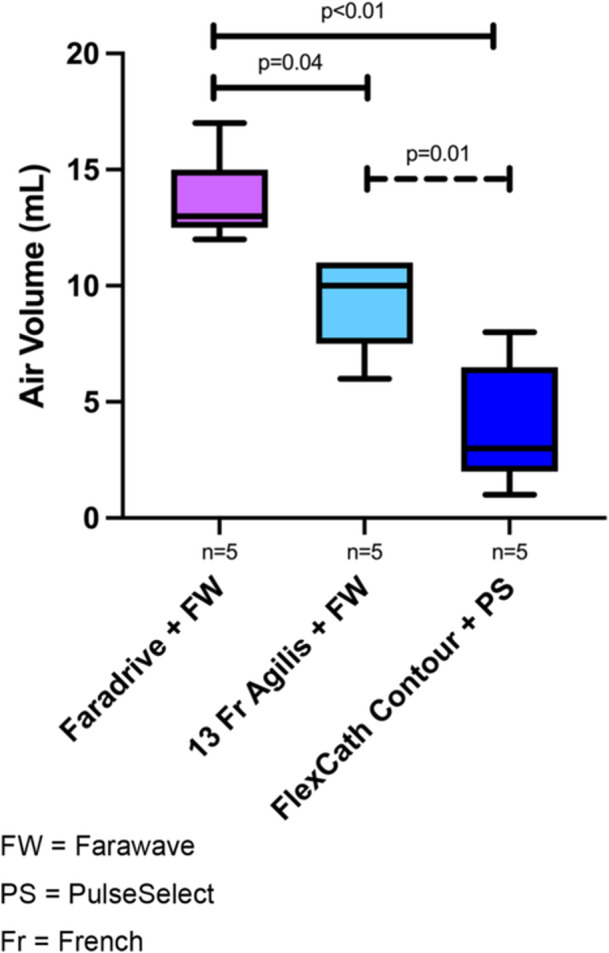
Box plots comparing average air intrusion volumes by PFA ablation system. A PFA ablation system includes a specific manufacturer's PFA catheter and PFA sheath combination. Each box plot represents the distribution of air intrusion volumes associated with a specific ablation system. Air intrusion was significantly higher when inserting the Farawave in the Faradrive sheath compared with inserting the Farawave in the 13 Fr Agilis NxT or the PulseSelect ablation catheter through the Medtronic FlexCath Contour. Fr, French; FW, Farawave; PS, PulseSelect.

A PFA “system” was defined as a manufacturer's PFA ablation catheter used with its corresponding sheath (e.g., the Boston Scientific Farawave ablation catheter with the Boston Scientific Faradrive sheath). Additionally, the Farawave catheter was also tested with the 13 Fr Agilis sheath. Air intrusion was significantly higher when inserting the Farawave in the Faradrive sheath compared with inserting the Farawave in the 13 Fr Agilis (13.6 + 2.0 mL vs. 9.4 + 2.1 mL, *p* = 0.04) or the PulseSelect ablation catheter through the FlexCath Contour (13.6 + 2.0 mL vs. 4.0 + 2.7 mL, *p* < 0.01). The Farawave catheter through the 13 Fr Agilis sheath led to significantly higher air intrusion compared with the PulseSelect ablation catheter through the FlexCath Contour (9.4 + 2.1 mL vs. 4.0 + 2.7 mL, *p* = 0.01) (Figure 3).

### 4.3 Mapping Versus Ablation Catheters

**Figure 4:**
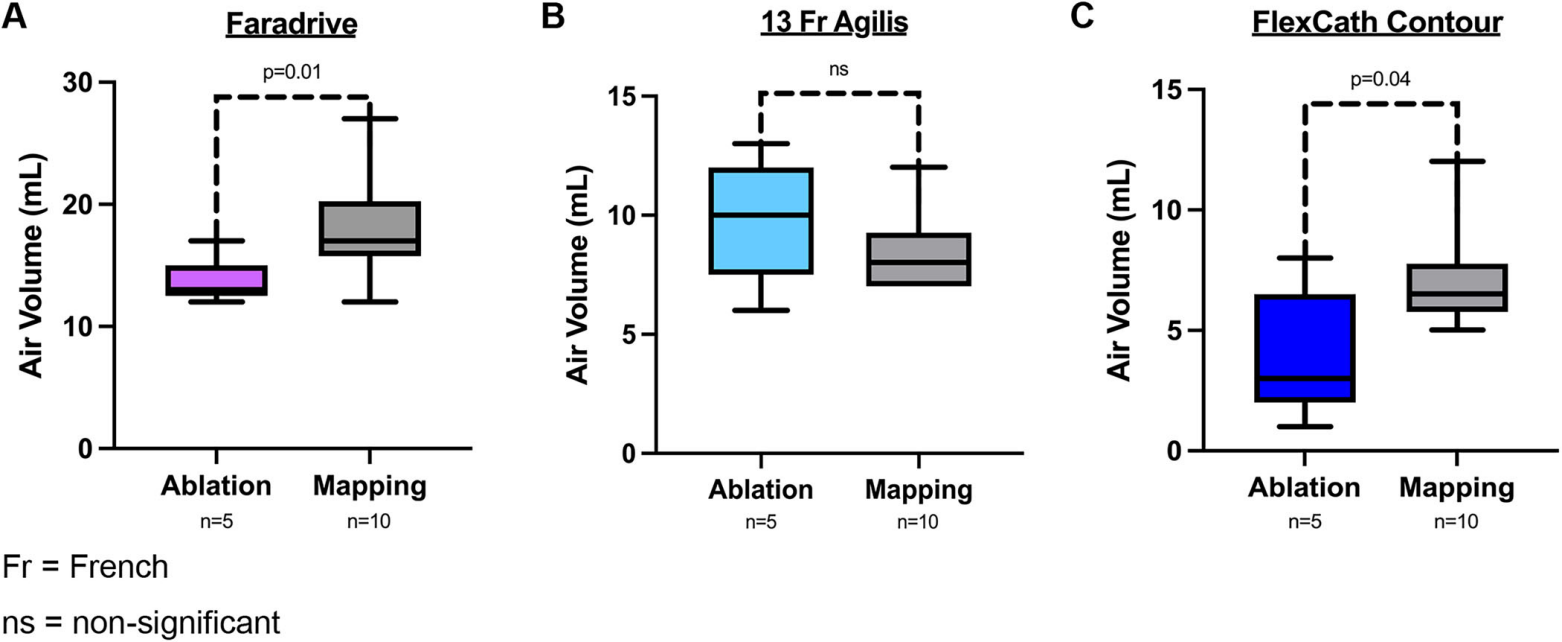
Box plots showing average air intrusion in each PFA sheath comparing mapping catheter to ablation catheter insertion. (A) Boston Scientific Faradrive. (B) Abbott 13 Fr Agilis NxT. (C) Medtronic FlexCath Contour. Each box plot represents the distribution of air intrusion volumes associated with an ablation or mapping catheter inserted into a specific sheath. A significantly higher volume of air intrusion was seen with mapping catheters compared with ablation catheters in both the Boston Scientific Faradrive and Medtronic FlexCath Contour sheaths. Fr, French; ns, nonsignificant.

A significantly higher volume of air intrusion was seen with mapping catheters compared with ablation catheters in both the Faradrive (18.0 ± 4.1 mL vs. 13.6 ± 4.1 mL, *p* = 0.03) and FlexCath Contour sheaths (7.1 ± 2.2 mL vs. 4.0 ± 2.7 mL, *p* = 0.04). There was no significant difference with the ablation catheter (Farawave) compared with mapping catheters in the 13 Fr Agilis (9.8 + 2.6 mL vs. 8.4 + 1.6 mL, *p* = 0.32) (Figure 4).

## 5 Discussion

To our knowledge, this is the first study to evaluate air intrusion into PFA ablation sheaths. Large volumes of air were entrained during ablation and mapping catheter insertion and removal. A significantly higher volume of air intrusion was seen using the Faradrive sheath compared with the FlexCath Contour, 13 Fr Agilis, and Vizigo. Additionally, larger volumes of air were seen with mapping catheter insertion when compared with ablation catheters.

Although the rate of air embolism in PFA studies has been low, undetected air intrusion may contribute to other adverse events [4]. The landmark MANIFEST‐17K study was a retrospective, observational study evaluating the safety of PFA using the Farapulse ablation catheter and Faradrive sheath in over 17 000 patients who underwent de novo AF ablation. Clinical air embolization was seen in 10 patients (0.06%). Additionally, there were 22 cerebrovascular accidents (CVAs), with four attributed to catheter exchange/sheath management. Sixteen of the patients who suffered CVAs underwent root cause analysis with no definitive cause identified in seven cases, suggesting that an alternative mechanism, such as air intrusion and resultant air emboli, may have contributed to the unexplained events [13].

Air intrusion into sheaths is likely influenced by multiple variables [3, 14]. However, three key factors may account for the differences observed in this study: sheath/catheter size mismatch, catheter tip shape, and the number and design of the sheath's hemostatic valve.

First, a greater sheath‐to‐catheter size mismatch likely contributes to increased air intrusion. In this study, a higher volume of air intrusion was observed in both the Faradrive and FlexCath Contour with mapping catheters compared with ablation catheters. There is a larger sheath‐to‐catheter mismatch between the large bore PFA sheaths and mapping catheters (8 Fr) compared with 12 Fr Farawave or 10 Fr PulseSelect ablation catheters, which likely resulted in higher volumes of air intrusion. However, despite a larger sheath‐to‐catheter mismatch, the 12 Fr FlexCath Contour with the 10 Fr PulseSelect led to less air intrusion than the Farawave through the Faradrive sheath or the Farawave through the 13 Fr Agilis, suggesting that factors beyond sheath‐to‐catheter mismatch contribute to air intrusion.

Second, each catheter evaluated in this study has a complex catheter tip shape, which may allow for more air intrusion during catheter insertion. To align with standard clinical practice, if a catheter introducer tool was present, it was filled with a column of water before insertion. Notably, the highest volume of air intrusion was seen with the Farawave catheter, which is the only catheter evaluated in this study that does not have an introducer tool. The lack of an introducer tool may limit the ability to eliminate air between splines of complex catheter designs by filling the column with fluid and lead to a higher volume of air intrusion.

**Figure 5:**
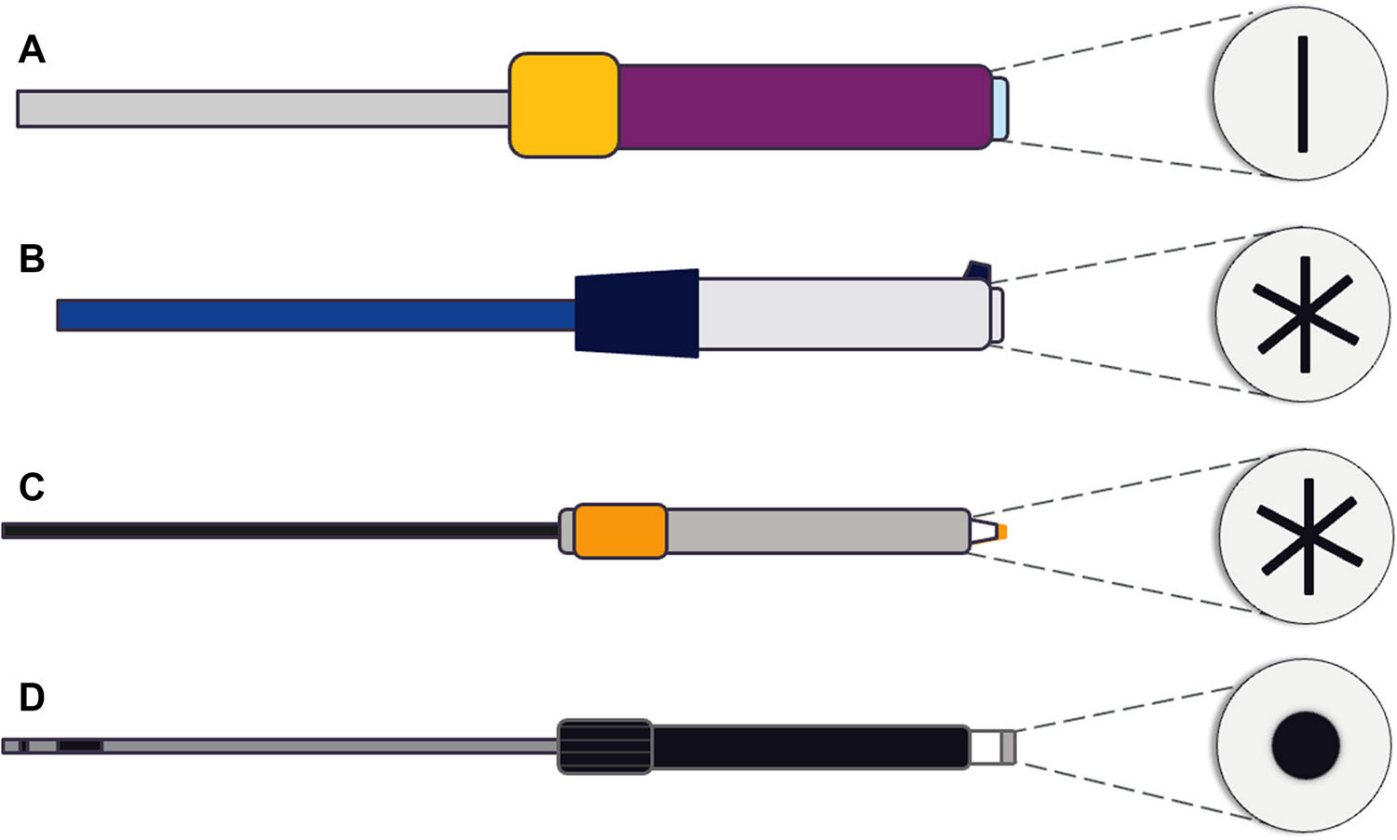
Sheath hemostatic valve opening designs. Each sheath has a unique hemostatic valve opening design that may contribute to differences in air intrusion. (A) Boston Scientific Faradrive has a cat‐eye opening. (B) Medtronic FlexCath Contour has an asterisk‐like opening. (C) Johnson & Johnson Medtech Vizigo has an asterisk‐like opening. (D) Abbott 13 Fr Agilis NxT has a round, recessed opening.

Finally, each sheath has been engineered with a proprietary hemostatic valve (or valves) to reduce air entry during catheter exchange. The differences in overall hemostatic valve construction, including valve shape, material, and number, may lead to differences in air intrusion. Externally, a notable difference is observed in the shape of the valve openings. The Faradrive sheath features a cat‐eye–shaped valve opening, the FlexCath Contour and Vizigo sheaths feature an asterisk‐shaped design, and the 13 Fr Agilis sheath features a round, recessed valve entry (Figure 5). This variation in the shape of valve opening may account for the differences in air intrusion observed. All sheaths tested feature a single hemostatic valve at the proximal hub, except for the 13 Fr Agilis, which includes an additional valve in the handle, which may further reduce air intrusion during catheter exchanges.

Two previous studies evaluated air intrusion into previous generation sheaths. Tsukahara and colleagues evaluated a cryoballoon sheath (FlexCath Advance; Medtronic) and a nonsteerable sheath (SL0; Abbott) and found that air intrusion volume was influenced by catheter tip shape, with thinner outer diameters causing greater sheath valve opening and increased air entry. In contrast to our study, they concluded that smaller caliber catheter shafts did not necessarily result in greater air intrusion compared with larger calibers [10]. However, the sheath/catheter mismatch may be sheath‐specific based on the interaction between catheter tip and sheath hemostatic valve design. Takami and colleagues evaluated air intrusion using a silicone heart model and high‐definition camera to quantify the amount of large or small air bubbles. They also evaluated the FlexCath Advance and SL0 sheaths, along with an 8.5 Fr Agilis (Abbott). They found air bubble intrusion was commonly observed during sheath flushing and catheter insertion, which varied by catheter type. Similar to Tsukahara's and the current study, they concluded that complex catheter tip shape may significantly contribute to air intrusion [11].

While improvements in sheath design have decreased air intrusion, complementary procedural techniques remain necessary as some degree of air intrusion during catheter exchange remains unavoidable. A number of methods to reduce the amount of air intrusion have been published. A “water seal method,” which involves immersing the sheath hub and catheter in saline during insertion, has been shown to reduce air entry [15]. Tsukahara and colleagues demonstrated that the “sheath‐in‐sheath” method, in which a catheter is advanced through a smaller sheath and then inserted inside a larger bore sheath, helped reduce air intrusion [10]. Additionally, Takami and colleagues demonstrated several techniques, including slow sheath flushing (5 mL/2 s vs. 15 mL/5 s) and temporary cryoballoon inflation before insertion (decreases the catheter–sheath size mismatch), helped reduce the volume of air entry into sheaths [11]. These studies emphasize adjunctive procedural strategies serve as a valuable complement to sheath design in limiting air intrusion.

The results of this study must be interpreted in light of its limitations. First, continuous negative pressure was applied, which does not mimic physiologic breathing patterns. Therefore, the model tested here may overestimate air intrusion, representing a worst‐case or maximal‐risk scenario. However, prior studies on air entry into ablation sheaths also used continuous negative pressure models. The absolute volume of air observed in this study was similar to that reported by Tsukahara and colleagues in trials using comparable sheath and catheter combinations that also created negative pressure via the siphon principle [10]. Second, the model in this study used water and may not reflect the effect of blood viscosity on air intrusion. Third, the volume of air intrusion measure may include air that entered through the hemostatic valve during withdrawal from the sidearm. Finally, this model lacked anatomical realism, including the important interaction between the sheath tip and myocardial tissue. If the sheath tip becomes occluded and a catheter is withdrawn, a vacuum can form, potentially leading to air intrusion. Some manufacturers have specifically designed their sheaths to help mitigate this vacuum effect when the tip is obstructed by tissue [6, 14].

## 6 Conclusion

A significantly higher volume of air intrusion was observed with the Faradrive sheath compared with the FlexCath Contour, 13 Fr Agilis NxT, and Vizigo sheaths. Insertion of mapping catheters led to higher volumes of air intrusion compared with ablation catheters in the Faradrive and FlexCath Contour sheaths. New sheath designs are needed to minimize air intrusion during catheter exchanges to avoid air embolism when performing PFA and using left atrial delivery sheaths.

## Conflicts of Interest

Dr. Aamir Ahmed has served as a consultant for Abbott and Medtronic. Dr. Susan S. Kim receives speaker honoraria from Medtronic and Abbott, and is on the advisory board for Boston Scientific. Dr. Bradley P. Knight receives speaker honoraria and consulting fees from Abbott, Biosense Webster, Boston Scientific, and Medtronic. Dr. Rod S. Passman receives research support from AHA (#18SFRN34250013), NIH (UG3HL165065), research support and speaker fees from Abbott, Boston Scientific, and Medtronic, and royalties from UpToDate. Dr. Kaustubha Patil receives speaker honoraria from Abbott and Zoll Medical. Dr. Nishant Verma receives speaker honoraria from Abbott, Biosense Webster, Boston Scientific, and Zoll. The other authors declare no conflicts of interest.

## Data Availability

Data that support the findings of this study are available from the corresponding author upon reasonable request.
